# Evidence for a Functional Link between Chromosomal Breakpoints and Aberrant DNA Methylation in Cancer

**DOI:** 10.3389/fonc.2014.00046

**Published:** 2014-03-11

**Authors:** Sven Bilke, Yevgeniy Gindin

**Affiliations:** ^1^Cancer Genetics Branch, Center for Cancer Research, National Cancer Institute, National Institutes of Health, Bethesda, MD, USA

**Keywords:** epigenetics, copy number aberrations, clonal evolution, mechanism, breast cancer

There has been a tremendous effort in systems biology to integrate multi-modal data in the hope to uncover and, ideally, provide a mechanistic explanation for the interconnectedness of the many features and aberrations of cancer genomes. A recent study authored by Tang and co-workers (1), which appeared in Frontiers in Cancer Genomics, provides an interesting example of this approach. It has now been “democratically” elected, based on the number of times that it has been accessed, to target the broader audience of Frontiers of Oncology.

In their work, Tang and colleagues use an interesting strategy to integrate DNA methylation and DNA copy number data from a curated set of 119 breast cancer samples leading to their central observation of a significant spatial association between differentially methylated regions and chromosomal breakpoints. Almost half (93) of 217 regions identified as differentially methylated by their in-house DNA methylation platform (MOMA) (2) were found to be located within 1 Mb of DNA breakpoint hot-spots, as assayed by their copy-number ROMA (3) platform for the same sample set. This observation of what they term Breakpoint Enriched Differentially Methylated Regions (BEDMRs) may be interpreted as indicative of a causal relation, whether direct or indirect, between DNA breaks and methylation. Such an association between hypomethylation and genomic instability has long been proposed (4) and an interesting mechanistic explanation has recently been offered by De and Michor (5). They observed that DNA copy-number aberrations are more likely to occur downstream of G4 quadruplex sequence motifs that contain hypo-methylated CpG di-nucleotides. The DNA molecule can locally, in the vicinity of such motifs, assume an alternate, non-B-form secondary structure, which can be an obstacle to replication forks (6) resulting in an increased chance of replication errors. The association between G4 quadruplexes and chromosomal aberrations may not hold in every case, however, as evidenced by Eriksson and colleagues (7) who report that this relationship exists in only some of the focal amplifications that they have analyzed.

Any model postulating a causal relationship between DNA breaks and aberrant methylation ultimately requires that both phenomena interact within the same cell. While the analysis performed by Tang and colleagues is compatible with this assumption, their strategy with its focus on “hot-spots” of DNA breaks and differential DNA methylation does not, necessarily, imply actual co-localization within any given cell. Also compatible with their analysis are models in which both phenomena are entirely unrelated on the level of individual events. In this case, the co-localization across the *set* of cancer samples could be the result of other mechanisms, such as selection during the clonal cancer evolution (8). The theoretical underpinning of this hypothesis is that both processes, independent of one another, contribute to dysregulation of gene expression in cancer. Copy number alterations are present in most cancers, frequently targeting cancer related genes (9) and display cancer subtype specific patterns that have been used for prognosis prediction, molecular sub-classification (10) and can even be used to develop tumor progression models (11). Similarly, DNA methylation, an important epigenetic regulator during normal cell development (12) and is altered in diseased cellular states (13). Patterns of aberrant methylation are a distinguishing feature of many cancer cells (4, 14) again leading to applications in prognosis prediction, classification, and diagnosis (15, 16). In their paper, Tang and co-workers investigate the tumor “clonal evolution” aspect by analyzing the gene content proximal to the BEDMRs. They find several cancer biology relevant genes, such as *AKT1, ARNT*, and *PMS2* and their literature analysis finds that 57% of the BEDMRs overlap with at least one gene that has more than three literature references related to cancer. Unfortunately, while this analysis is intriguing, the authors find that their results do not unequivocally prove the “tumor evolution” hypothesis, because the statistics do not quite reach the threshold of significance owing, likely, to their relatively small dataset of a heterogeneous disease – breast cancer.

Without overwhelming evidence in favor of a selection of cancer genes during clonal evolution, an alternative is that certain structural genomic elements might be particularly susceptible to both DNA breaks and aberrant methylation. The increased likelihood of recombination close to Alu and other genomic repetitive elements has been associated with an increased propensity for DNA breaks (17). At the same time, such repetitive elements are often GC rich, increasing the opportunity for differential methylation. In their study, Tang and colleagues report a statistically significant distance association between BEDMRs and Alu repeats within 3 Mb. Some 33 (35%) BEDMRs are located within 3 Mb of an Alu element. The majority of these BEDMRs (24/33) are hypo-methylated. These BEDMRs encompass a major histone cluster as well as a number of oncogenes.

The work by Tang et al. also looks at other facets of BEDMR biology. The authors’ analysis strongly hints that BEDMRs can be used to classify breast cancer tumors along the lines of gene expression-based subtypes. For instance, the authors identified 58 BEDMRs that are unique to basal-like samples. This observation agrees well with previous findings showing that expression-based breast cancer subtypes are clearly evident in DNA methylation (15) and copy-number (18) data. The authors suspect that a larger dataset is required to harness the power of BEDMRs to stratify breast cancer subtypes at a finer detail.

Going beyond the work presented by Tang and co-workers, expression analysis of genes located close to or within BEDMRs, which would be particularly sensitive to both methylation and copy number changes, could provide further evidence for selective pressure during cancer development shaping the spatial association in their target genes. If this is indeed the case, BEDMRs could be a stronger indicator for “interesting” genes and potential therapeutic targets than each signal individually. The strength, or probably more aptly “relative weakness,” of the association between BEDMRs and cancer related genes, however, suggests that the sample set size must be very large. In Tang’s work, the *p*-value barely reached the frequently used significance level of *p* < 0.05, and this only after “tuning” thresholds. In order to be successful, a study aiming to utilize a signal as weak as reported here would therefore have to involve a much larger number of samples, at *least* one order of magnitude or more. An extension of the analysis involving miRNA could be beneficial. A recent study examined the effect of DNA methylation and copy number alterations on the dysregulation miRNA expression in breast cancer, identifying 70 such miRNAs (19). Though Aure et al. used a different platform to assay DNA methylation and copy-number status, both they and Tang et al. relied, at least in part, on the same sample cohort (20).
